# FDG PET using SUV_max_ for preoperative T-staging of esophageal squamous cell carcinoma with and without neoadjuvant chemoradiotherapy

**DOI:** 10.1186/s12880-016-0171-7

**Published:** 2017-01-05

**Authors:** Yung-Cheng Huang, Hung-I Lu, Shun-Chen Huang, Chien-Chin Hsu, Nan-Tsing Chiu, Yu-Ming Wang, Yi-Chun Chiu, Shau-Hsuan Li

**Affiliations:** 1 Department of Nuclear Medicine, Kaohsiung Chang Gung Memorial Hospital and Chang Gung University College of Medicine, Kaohsiung, Taiwan; 2 Department of Thoracic and Cardiovascular Surgery, Kaohsiung Chang Gung Memorial Hospital and Chang Gung University College of Medicine, Kaohsiung, Taiwan; 3 Department of Pathology, Kaohsiung Chang Gung Memorial Hospital and Chang Gung University College of Medicine, Kaohsiung, Taiwan; 4 Department of Nuclear Medicine, National Cheng Kung University Hospital, College of Medicine, National Cheng Kung University, Tainan, Taiwan; 5 Department of Radiation Oncology, Kaohsiung Chang Gung Memorial Hospital and Chang Gung University College of Medicine, Kaohsiung, Taiwan; 6 Department of Hepato-Gastroenterology, Kaohsiung Chang Gung Memorial Hospital and Chang Gung University College of Medicine, Kaohsiung, Taiwan; 7 Department of Hematology-Oncology, Kaohsiung Chang Gung Memorial Hospital and Chang Gung University College of Medicine, Kaohsiung, Taiwan

**Keywords:** Esophageal cancer, Staging, Neoadjuvant chemoradiotherapy, Positron emission tomography

## Abstract

**Background:**

Accurate T-staging is pivotal for predicting prognosis and selecting appropriate therapies for esophageal squamous cell carcinoma (ESCC). The diagnostic performance of fluorodeoxyglucose (FDG) positron emission tomography/computed tomography (PET/CT) for its T-staging is uncertain. We investigated use of FDG PET/CT for preoperative T-staging of patients with ESCC.

**Methods:**

Patients with ESCC given preoperative FDG PET/CT scans, either with (CRT^[+]^ group) or without (CRT^[−]^ group) neoadjuvant chemoradiotherapy, were retrospectively reviewed. Maximal standardized uptake value (SUV_max_) of the primary tumors on FDG PET/CT scans were measured, and histopathological results were used as the reference standard. The associations between pathological T-stage and potential factors of age, tumor location, tumor grade, tumor size, and tumor SUV_max_ were analyzed. The cut-off levels of SUV_max_ for predicting different T-stages and for residual viable tumors after neoadjuvant chemoradiotherapy were determined using receiver operating characteristic analyses.

**Results:**

We enrolled 103 patients (45 in the CRT^[−]^ group; 58 in the CRT^[+]^ group). SUV_max_, an independent predictive factor, positively correlated with the pathological T-stage in both groups (CRT^[−]^ group: ρ = 0.736, *p* < 0.001; and CRT^[+]^ group: ρ = 0.792, *p* < 0.001). The overall accuracy of the PET/CT with thresholded SUV_max_ for predicting the pathological T-stage was 73.3% in the CRT^[−]^ group (SUV_max_ of T0: 0–1.9, T1: 2.0–4.4, T2: 4.5–6.5, T3: 6.6–13.0, T4: >13.0) and 67.2% in the CRT^[+]^ group (SUV_max_ of T0: 0–3.4, T1: 3.5–3.9, T2: 4.0–5.5, T3: 5.6–6.2, T4: > 6.2). For CRT^[−]^ group, the accuracy using an SUV_max_ cut-off of 4.4 to differentiate early (T0-1) from locally advanced disease (T2-4) was 82.2% (95% CI, 71.1–93.4%). For CRT^[+]^ group, the accuracy using an SUV_max_ cut-off of 3.4 to predict residual viable tumors (non-T0) after completion of chemoradiotherapy was 82.8% (95% CI, 73.0–92.5%).

**Conclusions:**

The FDG avidity of a primary esophageal tumor significantly positively correlated with the pathological T-stage. PET/CT with thresholded SUV_max_ was useful for predicting T-stage and differentiating residual viable tumors.

**Electronic supplementary material:**

The online version of this article (doi:10.1186/s12880-016-0171-7) contains supplementary material, which is available to authorized users.

## Background

Esophageal cancer, a poor prognostic disease with an estimated 5-year survival of 17–34%, occurs worldwide and is a leading cause of cancer mortality [1–3]. The two major histological types of esophageal cancer are squamous cell carcinoma and adenocarcinoma, which have different tumor biology and treatment outcomes [4]. With more sensitive to chemoradiation, esophageal squamous cell carcinoma (ESCC) has a higher complete response rate after neoadjuvant chemoradiotherapy (CRT) than adenocarcinoma [5]. In patients with resectable locally advanced ESCC, recent phase III study and meta-analysis have shown that neoadjuvant CRT followed by surgery is superior to surgery alone [6, 7]. After definitive CRT, around 32–46% of patients were free of viable tumors on the final surgical pathology examination [8, 9]. If tumors are totally eradicated after CRT, salvage esophagectomy predisposing to additional postoperative mortality (rate up to 10%) and morbidity (rate up to 50%) [10–12] may be unnecessary. Otherwise, surgery is suggested to eliminate local residual disease. To determine the most suitable therapy and to avoid inappropriate attempts at curative surgery, accurate preoperative T-stage and assessment of a patient’s response to CRT are required.

Endoscopic ultrasound (EUS) is considered the most accurate procedure for preoperative local staging of ESCC, but it is unreliable for staging after CRT largely due to the therapeutic related inflammatory effect or fibrosis [13, 14]. Moreover, EUS is relatively invasive and operator-dependent and it has two other limitations: severe stenosis blocks the passage of the endoscope, and its finite depth of penetration may be insufficient for staging T4 tumors.

For the initial staging and for evaluating a patient after CRT, the fluorodeoxyglucose (FDG) positron emission tomography/computed tomography (PET/CT) is useful for detecting lymphatic and hematogenous metastasis before surgery [15, 16]. But because of the limited spatial resolution, its role in classifying the T-stage is uncertain. For ESCC, there have been no established findings about the diagnostic ability of FDG PET/CT to predict the pathological T-stage. FDG avidity, semiquantitatively measured using standardized uptake values (SUVs) that reflect the aggressiveness of the neoplasm, can predict the extent of esophageal cancer [17, 18]. Some studies [18–20] have found associations between FDG avidity and T-stage, but the results are inconsistent, especially after neoadjuvant CRT. The routine use of FDG PET/CT for ESCC continues to grow. Beyond its well-known value in determining N and M stages, it is worth exploring its diagnostic performance for the T-stage. We investigated the application of FDG PET/CT for the preoperative T-staging of ESCC with and without neoadjuvant CRT.

## Methods

### Study design

We retrospectively reviewed consecutive ESCC patients who had undergone preoperative FDG PET/CT scans before the resection of their esophageal tumors in Chang Gung Memorial Hospital, Kaohsiung, Taiwan, between 2007 and 2013. Eligible patients were categorized into the CRT^[−]^ group, who did not undergo CRT for ESCC before resection, and the CRT^[+]^ group, who did and had FDG PET/CT after CRT. The CRT consisted of two cycles of 5-fluorouracil/cisplatin-based chemotherapy and thoracic radiation (3600 ~ 5040 cGy). All patients who underwent surgery had a radical esophagectomy with a cervical esophagogastrostomy or an Ivor Lewis esophagectomy with intrathoracic anastomosis, a two-field lymphadenectomy, reconstruction of the digestive tract with a gastric tube and pylorus drainage procedures. We used histopathological results as the reference standard. The pathological T-stages (T1-T4) were classified according to the 7th American Joint Committee on Cancer staging system [21]. For statistical analysis, high grade dysplasia (Tis) was classified as T0. The resected tissue was labeled by the surgeon and sent for pathological examination. The histopathological assessment was independently carried out by two pathologists, and a consensus was reached. For CRT^[+]^ group, pathologically complete response of primary tumor (T0) was defined as the complete disappearance of viable cancer cells in the tumor surgical specimens. Tumor histological grade (Gr1-Gr3) and tumor size (greatest dimension in cm) of the specimen as well as the initial biopsy location as proven ESCC via endoscopy were also recorded. We measured the maximal standardized uptake values (SUV_max_) of the primary esophageal tumors on FDG PET/CT scans. Multivariate analyses were used to evaluate the associations between pathological T-stage and the potential factors of age, tumor location, tumor grade, tumor size, and tumor SUV_max_. The cut-off levels of SUV_max_ for predicting different T-stages and for residual viable tumors after neoadjuvant CRT were determined using receiver operating characteristic (ROC) analyses. This retrospective study was approved by the Institutional Review Board of the Chang Gung Memorial Hospital with a waiver of consent.

### FDG PET/CT

After the patients had fasted for at least 6 h, they were injected with 370–555 MBq of FDG. PET/CT scans were initiated 1 h later using a combined PET/CT scanner (Discovery ST; GE Healthcare, Waukesha, WI, USA). For attenuation correction and imaging fusion, nonenhanced CT scans were acquired first from the mid-thigh to the head using the following parameters: 140 kV, 170 mA (maximum), and 3.75-mm thick sections. PET scans were then taken over the same anatomical regions for 5 min per bed position. The transaxial PET images were reconstructed using an ordered subsets expectation maximization algorithm in a matrix of 128 × 128-pixel with a slice thickness of 3.27 mm. The reconstructed images displayed in coronal and sagittal planes as well as maximum intensity projection images were also available for interpretation. SUVs were calculated according to the formula: SUV = measured activity within the region of interest (MBq/mL)/[injected dose of FDG (MBq)/body weight (g)]. The PET/CT images were reviewed and analyzed by a nuclear medicine physician (YCH, 7 years of experience in PET/CT reporting). Discrepancies with the routine PET/CT reports were resolved by consensus reviewing with a second nuclear medicine physician (CCH, 8 years of experience in PET/CT reading).

### Statistical analyses

Continuous variables were expressed as means with standard deviations (SD). Kolmogorov-Smirnov statistics were used to test the data sets for normal distribution. Student’s *t*-test was used in group comparisons of normally distributed data, and the Mann–Whitney *U*-test was used for data that were not normally distributed. Categorical variables were analyzed using the χ^2^ test. Potential factors associated with the pathological T-stage were identified separately in the CRT^[−]^ and the CRT^[+]^ group patients using ordinal logistic regression for multivariate analyses. The SUV_max_ of esophageal tumors in the 5 different pathological T-stages were compared using Kruskal-Wallis tests, and then analyzed using the Jonckheere-Terpstra test for ordered alternatives. The correlations between the SUV_max_ of esophageal tumors and pathological T-stages were analyzed using Spearman rank correlations. The cut-off levels of SUV_max_ for predicting different T-stages and for residual viable tumors in the CRT^[+]^ group were investigated using ROC analyses. The agreements between threshold-SUV_max_ and pathological T-stage were assessed from 5 × 5 tables using κ statistics. The κ values were classified as follows: ≤ 0.2, poor agreement; 0.21–0.4, fair agreement; 0.41–0.60, moderate agreement; 0.61–0.8, good agreement; and 0.81–1, excellent agreement. The sensitivity, specificity, positive predictive value (PPV), negative predictive value (NPV), and accuracy were calculated using standard formulas. SPSS 17 for Windows (SPSS Inc., Chicago, IL, USA) was used for all statistical analysis. Significance was set at *p* < 0.05.

## Results

**Fig. 1:**
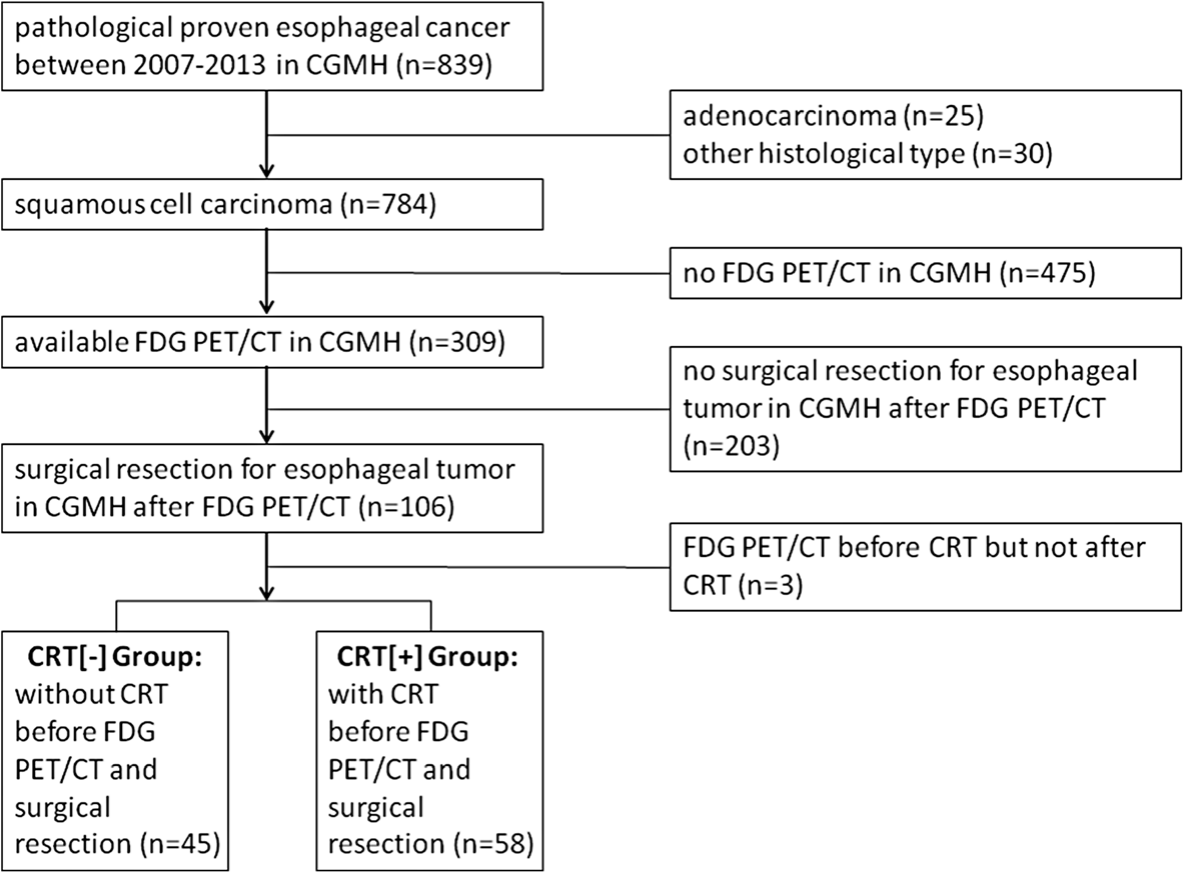
Flowchart of patients’ assessment for eligibility in the study

Of the 839 patients with ESCC that we reviewed, 103 eligible patients (100 men, 3 women; mean age: 53.6 ± 8.2 years old) were included in the analysis. The study flow chart with inclusion and exclusion criteria summarizes how the eligible patients were collected (Fig. 1). Forty-five patients had not undergone neoadjuvant CRT (the CRT^[−]^ group) and 58 patients had (the CRT^[+]^ group). The demographic features of the patients are summarized in Table 1. All of them had completed the FDG PET/CT scan. Their fasting blood sugar at scans was 102.2 ± 19.5 mg/dl (range: 61–175 mg/dl, CRT^[−]^ group: 96.6 ± 18.9 mg/dl; CRT^[+]^ group: 106.5 ± 19.0 mg/dl). Time from FDG injection to PET/CT scans was 61.7 ± 8.5 min (CRT^[−]^ group: 60.8 ± 7.6 min; CRT^[+]^ group: 62.4 ± 9.1 min). The FDG PET/CT scans did not identify the primary lesion in 2 of 2 patients with Tis lesions and 3 of 26 patients with T1 lesions in the CRT^[−]^ group or in the 1 patient with a Tis lesion and 2 of 4 patients with T1 lesions in the CRT^[+]^ group; they did, however, detect all the other primary tumors.

**Table 1:** Demographic and clinical characteristics of patients

Characteristic	Total (*n* = 103)	CRT^[−]^ group (*n* = 45)	CRT^[+]^ group (*n* = 58)
Age, years	53.6 (8.2)^a^	54.4 (8.0)^a^	53.1 (8.4)^a^
Gender (male:female)	100:3 (97%:3%)	44:1 (98%:2%)	56:2 (97%:3%)
Tumor location (upper:middle:lower)	25:52:26 (24%:51%:25%)	9:21:15 (20%:47%:33%)	16:31:11 (28%:53%:19%)
T-stage (T0:T1:T2:T3:T4)	28:30:14:20:11 (27%:29%:14%:19%:11%)	2:26:7:9:1 (4%:58%:16%:20%:2%)	26:4:7:11:10 (45%:7%:12%:19%:17%)
Tumor grade^b^ (G1:G2:G3)	4:60:11^b^ (5%:80%:15%)	2:36:5^b^ (5%:84%:11%)	2:24:6^b^ (6%:75%:19%)
Tumor size, cm	2.7 (1.4)^a^	2.7 (1.3)^a^	2.7 (1.5)^a^
Tumor SUV_max_	5.0 (3.0)^a^	5.3 (2.9)^a^	4.7 (3.0)^a^
CRT to PET/CT interval, days	N/A	N/A	52.2 (48.5)^a^
PET/CT to resection interval, days	24.4 (20.1)^a^	21.2 (20.9)^a^	26.8 (19.4)^a^

^a^Data are means (standard deviation)

^b^Tumor histological grade was not assessment in patients with resected tumor specimens classified as T0

*CRT* chemoradiotherapy, *PET*/*CT* positron emission tomography/computed tomography, *SUV*_max_ maximal standardized uptake value

The multivariate ordinal logistic regression analysis showed that both the SUV_max_ and tumor size were independent predictive factors of the pathological T-stage in the CRT^[−]^ group, but that the SUV_max_ was the only independent predictive factor of the pathological T-stage in the CRT^[+]^ group (Table 2).

**Table 2:** Multivariate analysis of the pathological T-stage

Parameter	Coefficient	95% confidence interval	*p* Value
*CRT* ^[−]^ *group*			
Age	0.066	−0.047 ~ 0.179	0.253
Tumor location^a^	0.748	−1.114 ~ 2.611	0.431
Tumor grade^b^	2.139	−1.393 ~ 5.671	0.235
Tumor size	0.715	0.038 ~ 1.392	0.038*
Tumor SUV_max_	0.894	0.400 ~ 1.389	<0.001*
*CRT* ^[+]^ *group*			
Age	−0.027	−0.108 ~ 0.053	0.504
Tumor location^a^	0.756	−0.955 ~ 2.468	0.386
Tumor grade^b^	−1.661	−3.814 ~ 0.493	0.131
Tumor size	0.206	−0.425 ~ 0.837	0.523
Tumor SUV_max_	1.111	0.530 ~ 1.692	<0.001*

^a^Lower vs. Upper + Middle

^b^Grade 3 vs. Grade 1 + 2. Tumor histological grade was not assessment in patients with resected tumor specimens classified as T0

*Statistically significant

*CRT* chemoradiotherapy, *SUV*_max_ maximal standardized uptake value

**Fig. 2:**
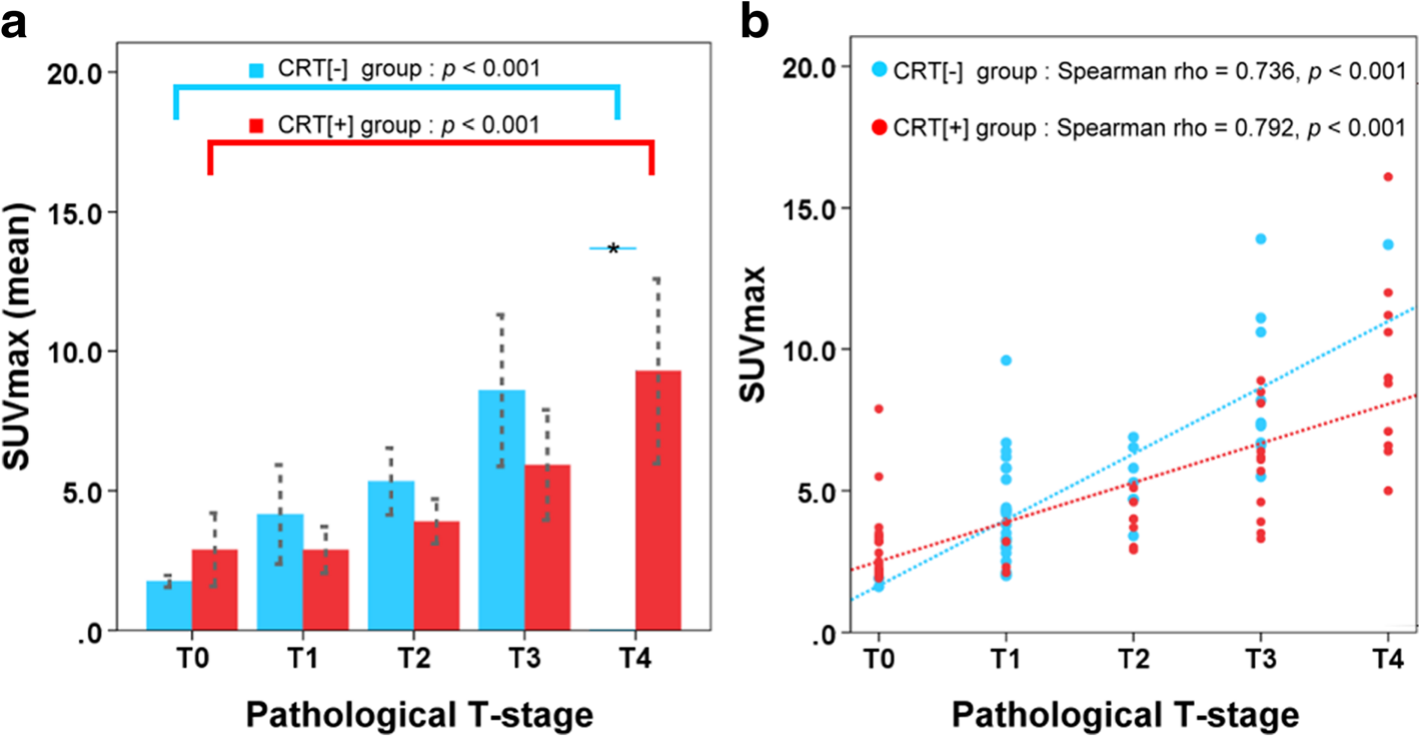
The relationships between the SUV_max_ of esophageal tumors and pathological T-stages. **a** Means of esophageal tumor SUV_max_ for the five pathological T-stages in the CRT^[−]^ group (*blue bars*) and the CRT^[+]^ group (*red bars*). A higher SUV_max_ was associated with a higher pathological T-stage (Jonckheere-Terpstra Trend Test across the 5 stage, all *p* < 0.001). Error bars were standard deviations. **b** The SUV_max_ of esophageal tumors were plotted against pathological T-stages in the CRT^[−]^ group (*blue dots*) and the CRT^[+]^ group (*red dots*). There were positive correlations between the tumor SUV_max_ and pathological T-stage (all *p* < 0.001)

The SUV_max_ between the five stages were significantly different between groups (Kruskal-Wallis Test; all *p* < 0.001), and a higher SUV_max_ was associated with a higher pathological T-stage (Jonckheere-Terpstra Trend Test across the 5 stages; all *p* < 0.001). There were positive correlations between the tumor SUV_max_ and the pathological T-stage (CRT^[−]^ group: ρ = 0.736, *p* < 0.001; and CRT^[+]^ group: ρ = 0.792, *p* < 0.001) (Fig. 2).

**Fig. 3:**
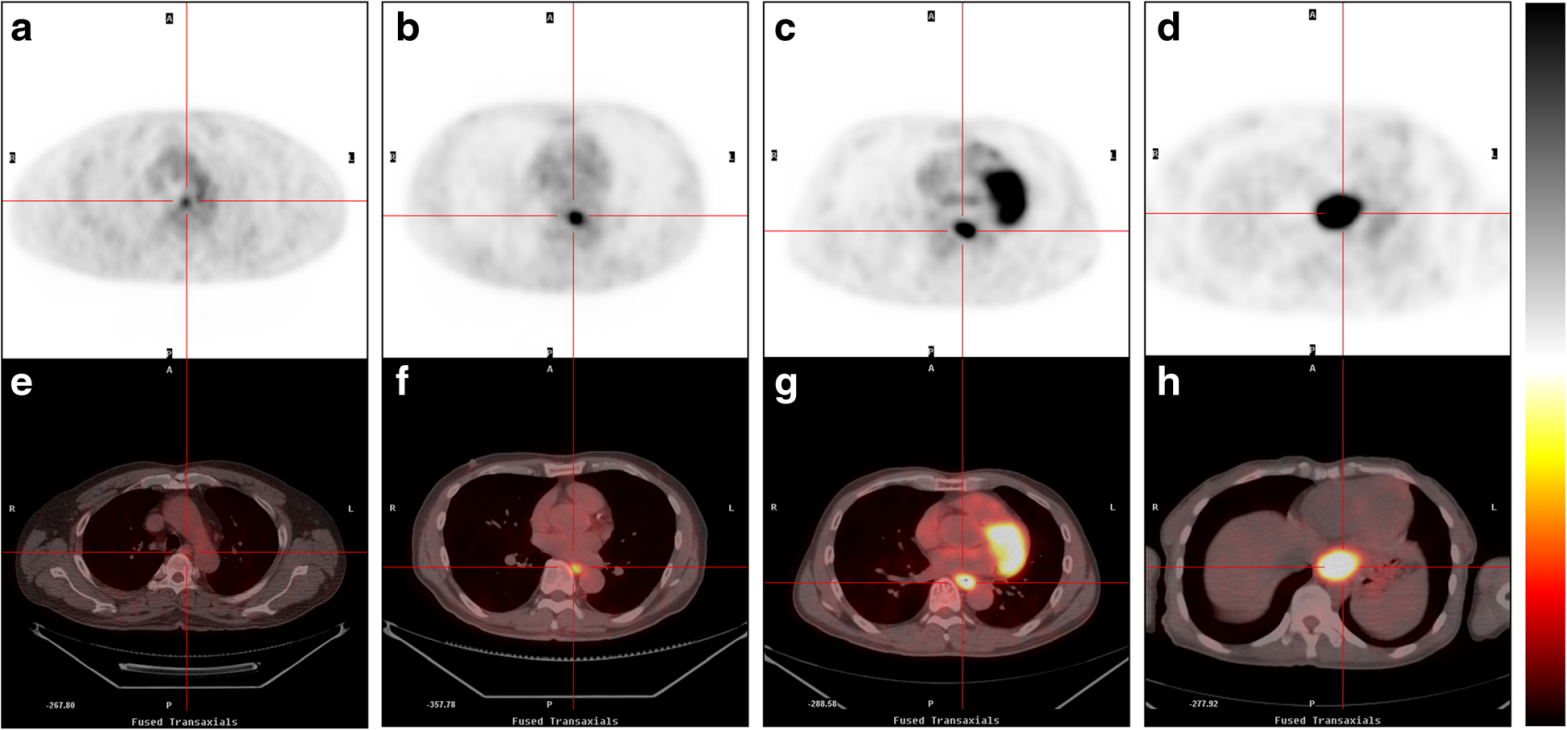
FDG PET (**a**–**d**) and corresponding fused PET/CT (**e**–**h**) transaxial images of four representative patients in the CRT^[−]^ group. Increased FDG uptake ranged from mild to intense in the pathologically proven T1 tumor with SUV_max_ = 3.5 (**a**, **e**); T2 tumor with SUV_max_ = 5.8 (**b**, **f**); T3 tumor with SUV_max_ = 8.2 (**c**, **g**); T4 tumor with SUV_max_ = 13.7 (**d**, **h**). The reference ranges of SUV_max_ cut-offs selected for CRT^[−]^ group were T1: 2.0–4.4, T2: 4.5–6.5, T3: 6.6–13.0, T4: >13.0

ROC curves for SUV_max_ were plotted to compare T0 vs. T1–4, T0–1 vs. T2–4, T0–2 vs. T3–4, and T0–3 vs. T4. According to the ROC curves, the ranges of SUV_max_ cut-offs selected were: for the CRT^[−]^ group = T0: 0–1.9, T1: 2.0–4.4, T2: 4.5–6.5, T3: 6.6–13.0, T4: >13.0; and for the CRT^[+]^ group = T0: 0–3.4, T1: 3.5–3.9, T2: 4.0–5.5, T3: 5.6–6.2, T4: > 6.2 (Table 3). For CRT^[−]^ group, the accuracy of differentiating early (T0–1) from locally advanced disease (T2–4) was 82.2% (95% CI, 71.1–93.4%). Representative cases of the FDG uptake in pathological T1–T4 esophageal tumors are shown in Fig. 3. Using PET/CT with thresholded SUV_max_, the T-stage was overstaged for 9 of the patients (20.0%) and understaged for 3 (6.7%) in the CRT^[−]^ group; and it was overstaged for 8 of the patients (13.8%) and understaged for 11 (19.0%) in the CRT^[+]^ group. The overall accuracy of the thresholded SUV_max_ for predicting pathological T-stage were 73.3% (κ = 0.628, good agreement) in the CRT^[−]^ group and 67.2% (κ = 0.538, moderate agreement) in the CRT^[+]^ group (Table 4).

**Table 3:** Determination of SUV_max_ cut-offs for pathological T-stage

T-stage	SUV_max_ cut-off	AUROC curve	95% confidence interval
*CRT* ^[−]^ *group*			
T ≥ T1	1.9	1.00^a^	0.92 ~ 1.00
T ≥ T2	4.4	0.88^a^	0.75 ~ 0.96
T ≥ T3	6.5	0.95^a^	0.84 ~ 0.99
T : T4	13.0	N/A^b^	N/A
*CRT* ^[+]^ *group*			
T ≥ T1	3.4	0.89^a^	0.77 ~ 0.95
T ≥ T2	3.9	0.93^a^	0.83 ~ 0.98
T ≥ T3	5.5	0.95^a^	0.85 ~ 0.99
T : T4	6.2	0.95^a^	0.86 ~ 0.99

^a^ *p* < 0.05

^b^Data unavailable because there was only one patient with a T4 tumor in the CRT^[−]^ group

*AUROC* area under the receiver-operating-characteristic, *CRT* chemoradiotherapy, *SUV*_max_ maximal standardized uptake value

**Table 4:** Diagnostic performance of PET/CT using thresholded SUV_max_

	Pathological T-stage				
	T0	T1	T2	T3	T4
*CRT* ^[−]^ *group*	2	26	7	9	1
PET/CT					
T0	**2**	1			
T1		**18**	1		
T2		5	**5**	1	
T3		2	1	**7**	
T4				1	**1**
Accuracy = 73.3% (33/45)					
*CRT* ^[+]^ *group*	26	4	7	11	10
PET/CT					
T0	**22**	3	2	1	
T1	2	**1**	1	2	
T2			**4**	1	1
T3	1			**3**	
T4	1			4	**9**
Accuracy = 67.2% (39/58)					

*CRT* chemoradiotherapy, *PET*/*CT* positron emission tomography/computed tomography

To detect residual viable tumor (non-T0) after CRT, the optimal SUV_max_ cut-off was 3.4 with sensitivity of 81.3% (95% CI, 63.0–92.1%); specificity of 84.6% (95% CI, 64.3–95.0%); PPV of 86.7% (95% CI, 68.4-95.6%); NPV of 78.6% (95% CI, 58.5–90.1%); and accuracy of 82.8% (95% CI, 73.0–92.5%). The area under the ROC curve was 0.89 (95% CI, 0.77–0.95, *p* < 0.001). Setting the SUV_max_ cut-off value at 2.2 with a negative likelihood ratio of 0.07 reduced the false-negative rate to 3.1% (sensitivity: 96.9%, specificity: 46.2%). Setting it at 5.5 with a positive likelihood ratio of 13.00 reduced the false-positive rate to 3.9% (sensitivity: 50%, specificity: 96.1%).

## Discussion

Our study showed that SUV_max_ of the esophageal tumor was the most significant independent factor associated with the pathological T-stage. Furthermore, using ROC analysis to define SUV_max_ cut-offs, we found that FDG PET/CT was able to predict pathological T-stage with acceptable accuracy (CRT^[−]^ group: 73.3%; CRT^[+]^ group: 67.2%). Using an SUV_max_ cut-off of 4.4 to differentiate early (T0-1) from locally advanced disease (T2–4) for CRT^[−]^ group and an SUV_max_ cut-off of 3.4 to predict residual viable tumors (non-T0) for CRT^[+]^ group, yielded an optimal diagnostic accuracy of 82.2% and 82.8%, respectively. These results indicated FDG PET/CT may provide preoperative T-staging of ESCC.

The SUV_max_ is a widely accepted and feasible parameter of PET/CT image used for cancer diagnosis and disease evaluation [17, 20, 22–25] because it is less observer-dependent and more reproducible than SUV_mean_ [26]. There must be variation in SUV_max_ used among different institutions with different PET/CT equipment. Our data were derived from the same PET/CT scanner with standardized image acquisition and reconstruction protocol that minimized the variation of technological factors. The other biological factors including radiotracer distribution time and level of fasting blood glucose were within a limited range, which would reduce variability in our SUV data. Another parameter of FDG PET, the total lesion glycolysis (TLG), is defined as the product of SUV_mean_ and metabolic tumor volume (MTV). The TLG values are believed to reflect both the biological aggressiveness and tumor burden. Although data are limited with respect to TLG and esophageal cancer, recent reports suggest that TLG may be a useful prognostic factor [27, 28]. However, the lesions are usually defined by using a threshold method and thus the setting of threshold will result in some degree of variations in SUV_mean_, MTV and TLG. Currently, no single optimal threshold can provide accurate tumor delineation. Further validation of different published methods for measuring the tumor volumes is still needed.

PET/CT is known to have a limited role in evaluating an early-stage cancer with small volume and its T descriptor because of its restrictive spatial resolution. Using CT anatomical information and knowledge of the distance from the incisors to the tumor being endoscopically viewed, we could identify the ESCC for 25 of 30 patients (83.3%) with T1 tumors on PET/CT scans, even though it could not identify Tis tumors. The detection rate was much higher than that obtained using a PET scanner alone, which ranged from 43 to 55% [29, 30], and slightly higher than the 71% for T1 tumors reported by Manabe et al. [20], who used a PET/CT scanner.

Sun et al. [31] reported that tumor length affects FDG uptake in esophageal cancer, and that the T-stage of the primary tumor is not significantly correlated with the SUV_max_ after controlling for length. However, instead of multiplying the number of slices by the slice thickness on PET scans for tumor length and using clinical T-stage for the reference standards as they did, we used the pathological tumor size and pathological T-stage of the specimens as reference standards to permit a more reliable evaluation.

In the CRT^[−]^ group, the major mistake made by PET/CT with thresholded SUV_max_ was overstaging, which accounted for 75% (9/12) of the erroneously staged patients. Six of the nine overstaged patients had polypoid tumors. A polypoid tumor protruding into the esophageal lumen but not aggressively extended through the esophageal wall might show high FDG avidity without being at an advanced T-stage. A representative example is shown in the additional file (Additional file 1: Figure S1). Using thresholded SUV_max_ to predict pathological T-stage, we need to be aware of the pitfall that polypoid tumors might have high SUV_max_ and thus can be easily overstaged. PET/CT does not offer detail anatomic information on how deeply the tumor has grown into the esophageal wall or into nearby structures. Other alternatives such as PET/MR [32] or EUS if feasible may offer superior resolution to get more reliably T stage for esophageal cancer. In patients without luminal obstruction or patients had no prior CRT before surgery, the T stage should be determined according to the EUS for increased accuracy [33].

Several studies [25, 34–36] have documented the value of a PET scan for assessing the esophageal tumor response to neoadjuvant CRT and for helping to identify residual disease after CRT. The PET scan, which is characterized by measuring the FDG concentration in metabolically active tissue, is independent of morphology and size despite a persistent mass effect, and offers the advantage of allowing us to differentiate viable tumors from tissue with no residual cancer. Using the qualitative or quantitative imaging analysis of the PET scan, the accuracy of the predictive value compared with the final pathology examination finding was reported to range between 53 and 79% [25, 34–36]. In the meta-analysis of Kwee et al. [37], they recommended that FDG PET should not yet be used in routine clinical practice to guide neoadjuvant therapy decisions. This conclusion was made from twenty heterogeneous groups with heterogeneous analysis methods. Most of them were composed with both adenocarcinoma and squamous cell carcinoma. The largest group with pure squamous cell carcinoma enrolled 32 patients with ESCC [25]. In a subgroup composed of 20 patients, they found the metabolic response measured by SUV_max_ changes between pre-CCRT and post-CCRT FDG PET scans were related to pathologic response with an accuracy of 70% [25]. Our current study provided a larger group of patients with ESCC adds to the numerous studies that have already been performed. In our ROC analysis, the optimal cut-off SUV_max_ of 3.4 for a residual viable tumor was acceptably accurate: 82.8%. Moreover, an extremely low false-negative rate of 3.1% would be reached by setting the SUV_max_ cut-off at 2.2, which is comparable to the expected mortality rate of an esophagectomy; and an extremely low false-positive rate of 3.9% would be reached by setting the SUV_max_ cut-off at 5.5. These data may help physicians to provide opinions for patients with different comorbidities or operative risks to consider suspending or undergoing an esophagectomy after neoadjuvant CRT.

This study has several limitations. It was retrospective and thus prone to a selection bias. There was an unequal number of patients among each stage. Because of T4 lesion was not suitable for surgery without preoperative CRT in previous clinical practice, there was only one patient with a T4 lesion in the CRT^[−]^ group. Moreover, the variance in SUV values of different PET/CT equipment and the test-retest reproducibility were unavailable from our data. These results should be validated in a prospective trial of FDG PET/CT for esophageal cancer.

## Conclusions

FDG avidity of a primary esophageal tumor significantly positively correlated with the pathological T-stage. Although inherently unable to provide sufficient data directly distinguish how deeply the tumor has grown into the esophageal wall, PET/CT with thresholded SUV_max_ is useful for predicting the T-stage and for differentiating residual viable tumors of ESCC, which has the potential helpfulness to select treatment strategies for patients with esophageal cancer.

## Additional file

Additional file 1: Figure S1. One patient in the CRT^[−]^ group with polypoid esophageal cancer and unexpectedly high fluorodeoxyglucose (FDG) avidity. Post-surgical histopathology proved the tumor to be stage T1. Representative (a) coronal (b) sagittal, and (c) transaxial computed tomography (CT), positron emission tomography (PET), and PET/CT fusion images (from left to right) showed a focal area of intensely increased FDG uptake in the lower thoracic esophagus (maximal standardized uptake value = 9.6); (d) endoscopy showed a polypoid tumor at about the 35 cm level from central incisors; and (e) endoscopic ultrasound showed the focal mucosal polypoid tumor (14.9 mm × 13.5 mm) invading the muscularis propria. (PDF 666 kb)

## Abbreviations

CRT: Chemoradiotherapy
ESCC: Esophageal squamous cell carcinoma
EUS: Endoscopic ultrasound
FDG: Fluorodeoxyglucose
MTV: Metabolic tumor volume
NPV: Negative predictive value
PET/CT: Positron emission tomography/computed tomography
PPV: Positive predictive value
ROC: Receiver operating characteristic
SD: Standard deviations
SUV_max_: Maximal standardized uptake value
SUVs: Standardized uptake values
TLG: Total lesion glycolysis
