# Supplementary material for: FDG PET using SUVmax for preoperative T-staging of esophageal squamous cell carcinoma with and without neoadjuvant chemoradiotherapy
Source: BMC Med Imaging. 2017 Jan 5;17:1. doi: 10.1186/s12880-016-0171-7 (PMC5217536; doi:10.1186/s12880-016-0171-7)
Supplement: Additional file 1: Figure S1. — One patient in the CRT[−] group with polypoid esophageal cancer and unexpectedly high fluorodeoxyglucose (FDG) avidity. Post-surgical histopathology proved the tumor to be stage T1. Representative (a) coronal (b) sagittal, and (c) transaxial computed tomography (CT), positron emission tomography (PET), and PET/CT fusion images (from left to right) showed a focal area of intensely increased FDG uptake in the lower thoracic esophagus (maximal standardized uptake value = 9.6); (d) endoscopy showed a polypoid tumor at about the 35 cm level from central incisors; and (e) endoscopic ultrasound showed the focal mucosal polypoid tumor (14.9 mm × 13.5 mm) invading the muscularis propria. (PDF 666 kb) [file 12880_2016_171_MOESM1_ESM.pdf]

Supporting Data

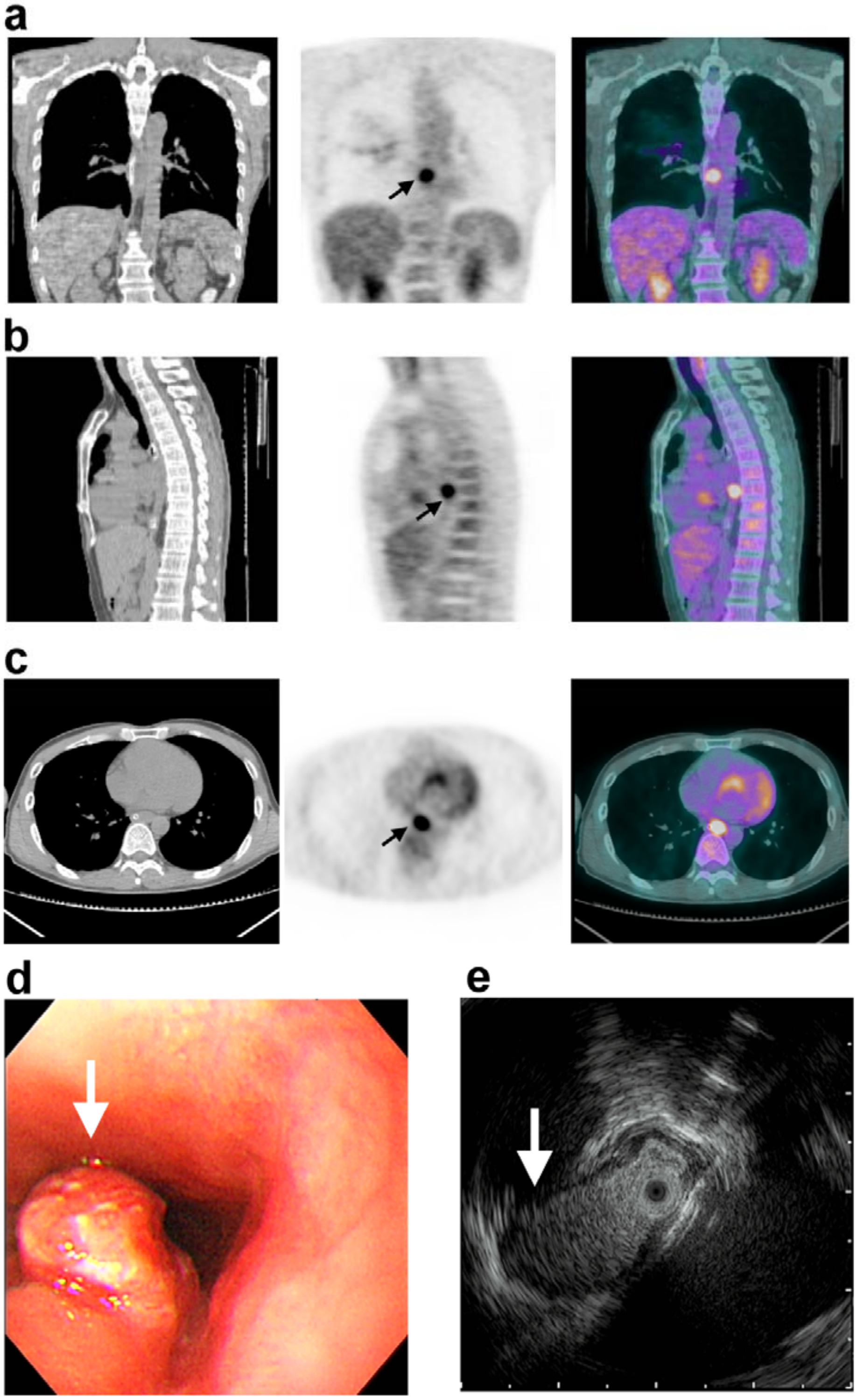

Figure S1. One patient in the CRT[−] group with polypoid esophageal cancer and unexpectedly high fluorodeoxyglucose (FDG) avidity. Post-surgical histopathology proved the tumor to be stage T1. Representative coronal (a), sagittal (b), and transaxial (c) computed tomography (CT), positron emission tomography (PET), and PET/CT fusion images (from left to right) showed a focal area of intensely increased FDG uptake in the lower thoracic esophagus (maximal standardized uptake value = 9.6); (d) endoscopy showed a polypoid tumor at about the 35cm level from central incisors; and (e) endoscopic ultrasound showed the focal mucosal polypoid tumor (14.9mm × 13.5mm) invading the muscularis propria.
